# CD169 (Siglec-1) as a Robust Human Cell Biomarker of Toll-Like Receptor 9 Agonist Immunotherapy

**DOI:** 10.3389/fcimb.2022.919097

**Published:** 2022-07-05

**Authors:** Stine Sofie Frank Lende, Marie Høst Pahus, Ida Monrad, Rikke Olesen, Anna R. Mahr, Line K. Vibholm, Lars Østergaard, Ole Schmeltz Søgaard, Anna Halling Folkmar Andersen, Paul W. Denton, Martin Tolstrup

**Affiliations:** ^1^ Department of Clinical Medicine, Aarhus University, Aarhus, Denmark; ^2^ Department of Infectious Diseases, Aarhus University Hospital, Aarhus, Denmark; ^3^ Department of Biology, University of Nebraska at Omaha, Omaha, NE, United States

**Keywords:** biomarker, CD169, toll-like receptor 9 (TLR9), immunotherapy, HIV-1

## Abstract

Immunotherapy is a promising therapeutic area in cancer and chronic viral infections. An important component of immunotherapy in these contexts is the activation of innate immunity. Here we investigate the potential for CD169 (Siglec 1) expression on monocytes to serve as a robust biomarker for activation of innate immunity and, particular, as a proxy for IFN-α production. Specifically, we investigated the effects of *Toll*-like receptor 9 agonism with MGN1703 (lefitolimod) across experimental conditions *ex vivo*, in humanized mice, and in clinical trial participants. *Ex vivo* we observed that the percentage of classical monocytes expressing CD169 increased dramatically from 10% pre-stimulation to 97% 24 hrs after MGN1703 stimulation (p<0.0001). In humanized NOG mice, we observed prominent upregulation of the proportions of monocytes expressing CD169 after two doses of MGN1703 where 73% of classical monocytes were CD169 positive in bone marrow following MGN1703 treatment vs 19% in vehicle treated mice (p=0.0159). Finally, in a clinical trial in HIV-infected individuals receiving immunotherapy treatment with MGN1703, we observed a uniform upregulation of CD169 on monocytes after dosing with 97% of classical monocytes positive for CD169 (p=0.002). Hence, in this comprehensive evaluation *ex vivo*, in an animal model, and in a clinical trial, we find increases in the percentage of CD169 positive monocytes to be a reliable and robust biomarker of immune activation following TLR9 agonist treatment.

## Introduction

The HIV-1 pandemic remains at a high level with 1.5 million new annual HIV-1 infections. Despite very effective antiretroviral therapy (ART) that blocks disease progression, those who become infected with HIV-1 currently face life-long adherence to ART as a means to control the infection. This is because there is not yet a large-scale curative intervention. Many efforts are being put into strategies that aim to deliver HIV-1 remission or cure. Among these are boosting existing or inducing *de novo* immune responses targeting HIV-1 (Martinsen et al., 2020). In such immunotherapy approaches, there is a great focus on the innate immune system because of its important role in the early defense against infections. Endogenous interferon (IFN) production induced by activation of *Toll*-like receptors (TLRs) is one of the innate immune system’s first antiviral responses upon infection (Mogensen, 2009). TLRs are pattern recognition receptors (PRRs) which detect pathogen-associated molecular patterns (PAMPs) (Kawai and Akira, 2010). TLRs are expressed on a multitude of immune cells including natural killer (NK) cells, macrophages, B cells, and to a high degree on dendritic cells (DCs) - including plasmacytoid dendritic cells (pDCs) (Hornung et al., 2002). TLR1, -2, -4, -5, -6, and -10 are expressed on the cell surface, whereas TLR3, -7, -8, and -9 are located in the membrane of endosomes. This partitioning in TLR localization reflects the disparate pathogen-sensing function of these important PRRs. The cell surface-associated TLRs are mainly responsible for detecting components from extracellular microbes such as bacteria and fungi. In contrast, the TLRs in the endosomal compartment mainly detect nucleic acids from virus and intracellular bacteria (Thompson and Iwasaki, 2008; Iwasaki and Medzhitov, 2015). The cytokine induction initiated by TLR activation not only triggers an innate immune response, but also takes part in initiating and shaping the adaptive immune response. Therefore, agonists of the TLR system have been extensively developed as vaccine adjuvants and as components of immunotherapy (Krieg, 2006; Falloon et al., 2016; Ribas et al., 2021).

MGN1703 (lefitolimod), a novel TLR9 agonist, belongs to a class of drugs referred to as immune surveillance reactivators and has been tested in phase 3 for treatment of metastatic colorectal cancer (NCT02077868). Pre-clinical testing, and data from cancer treatment trials, demonstrates that MGN1703 activates immune functions through the TLR9 pathway (Schmoll et al., 2014; Schmidt et al., 2015). TLR9 agonism by MGN1703 leads to the secretion of a range of cytokines (e.g., IFN-α) and to an upregulation of activation markers on pDCs and B cells (Schmoll et al., 2014; Schmidt et al., 2015; Weihrauch et al., 2015; Wittig et al., 2015). We recently demonstrated, *ex vivo* and *in vivo*, that MGN1703 dosing in HIV-1 infected individuals enhanced their cellular immune responses, increased HIV-1 production in CD4^+^ T cells, and primed a strong interferon-induced antiviral transcriptional program (Offersen et al., 2016; Vibholm et al., 2017; Krarup et al., 2018; Schleimann et al., 2019; Vibholm et al., 2019). Thus, MGN1703 may confer a dual effect in HIV-1 eradication strategies by reversing latency as well as enhancing cellular immune responses altogether.

Siglecs are cell-surface, sialic acid-binding receptors located on cells of hematopoietic lineage (Chang and Nizet, 2014; Chang and Nizet, 2020). “Siglec” is an abbreviation of sialic acid-binding immunoglobulin-type lectin. CD169 (Siglec-1) is the first Siglec described in this receptor family that comprise at least 14 human members. CD169 is primarily expressed on monocyte/macrophages and does not contain an intracellular signal domain. Thus, CD169 is believed to primarily be involved in cell-cell and cell-microbe interactions. CD169 exhibits important phagocytic functions, including facilitating antigen uptake, and is highly expressed on myeloid cells in draining lymph nodes following infections (Crocker et al., 1997) and was recently shown to be a prognostic factor in malignant melanoma and as a biomarker of SARS-CoV-2 infection (Saito et al., 2015; Bedin et al., 2021). In line with these observations, CD169 is highly inducible by type I IFN (e.g., IFN-α) and thus CD169 forms a natural link between innate immune activation and the role in antigen scavenging and cross-presentations functions in formation of adaptive immune responses (Puryear et al., 2013).

Given that type I IFN induces CD169 expression on human cells *ex vivo* (Puryear et al., 2013) combined with our data demonstrating *in vivo* production of IFN-α following MGN1703 dosing in trial participants (Offersen et al., 2016; Vibholm et al., 2017; Krarup et al., 2018; Schleimann et al., 2019; Vibholm et al., 2019), we investigated whether MGN1703 upregulated CD169 expression *ex vivo*, in humanized mice and in humans within a clinical trial setting. Our goal for this exploration was to determine whether changes in CD169 expression in response to TLR9 agonist (MGN1703 in particular) immunotherapy could serve as an easy-to-measure and reliable biomarker for verifying immune activity. This is particularly important because IFN-α can be challenging to measure directly in *in vivo* samples that are typically available in limited quantities. Here we present data demonstrating that CD169 is a powerful and sensitive biomarker of TLR9 agonist bioactivity.

## Results

### TLR9 Agonist Stimulation Ex Vivo

**Figure 1:**
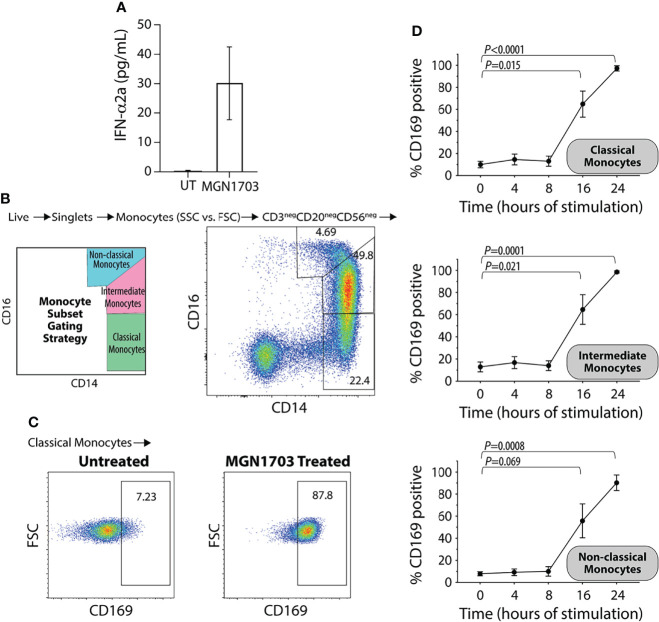
Upregulation of CD169 on human monocytes following *ex vivo* TLR9 agonist stimulation. **(A)** Levels of IFN-α in supernatants from human PBMCs stimulated with TLR9 agonist (MGN1703 3μM) for 24 hrs. Bar-graph depicts average IFN-α levels obtained from stimulation of five individual donors (+/- SEM). UT=untreated. **(B)** Flow plot demonstrating monocyte subset gating strategy with representative data. Classical monocytes CD14+/CD16^int^, Intermediate monocytes CD14+/CD16+ and the non-classical monocytes CD14^int^/CD16+. **(C)** Flow plots depicting the impact of MGN1703 on CD169 expression on classical monocytes after 24 hrs of stimulation for a representative donor **(D)** Time-dependent upregulation of CD169 on monocytes. Human PBMCs were stimulated with TLR9 agonist (MGN1703 at 3 μM) for 4, 8, 16 or 24 hrs. Levels of CD169 on monocyte subsets were measured using flow cytometry. Depicted in the graph is the average (+/- SEM) from five individual donors. Levels of CD169 on each monocyte subset were analyzed by paired ANOVA and Dunnett’s multiple comparison tests were performed for each time-point compared to baseline.

We have previously shown that stimulation of human PBMCs with MGN1703 leads to clear and strong production of IFN-α (Offersen et al., 2016; Vibholm et al., 2017). Further, previous studies have shown that recombinant IFN-α induce CD169 upregulation on monocyte-derived macrophages and TLR9 agonism (CpG-ODN 2216) specifically, can upregulate CD169 expression on a total PBMC population (York et al., 2007; Saito et al., 2015). Therefore, we first evaluated whether MGN1703 immunotherapy would induce increased CD169 levels on primary human monocytes *ex vivo*. At 24 hours post-stimulation, 3 µM MGN1703 led to secretion of IFN-α in cell culture supernatants (**Figure 1A**; mean 29.4 pg/mL from five independent donors). Next, we investigated the upregulation of CD169 on monocytes following MGN1703 stimulation using flow cytometry. In **Figures 1B, C** the classification scheme of monocytes as classical (CD14+/CD16-), intermediate (CD14+/CD16^int^) or non-classical (CD14^int^/CD16+) is shown together with representative data demonstrating a shift in the proportion of classical monocytes expressing CD169 following MGN1703 exposure. Specifically, without stimulation, only 7.23% of the monocytes were positive for CD169 cell-surface expression. The percentage of classical monocytes exhibiting CD169 cell-surface expression increased to 87.8% following treatment with the TLR9-agonist MGN1703. **Figure 1D** shows the time-dependent effect of the TLR9-agonist-induced upregulation of CD169 on all three subsets of monocytes during PBMC incubation with MGN1703. Throughout the first 8 hours of incubation, the levels of monocytes expressing CD169 remained unchanged with a percentage of CD169 positive monocytes at baseline at 10.1%, 12.9% and 7.9% for classical -, intermediate- and non-classical monocytes, respectively. By 16 hours post-stimulation with MGN1703, the percentage of CD169 positive monocytes increased significantly. This was true for all monocyte subsets. By 24 hours post-stimulation, at least 90% of all monocytes analyzed were positive for CD169 (i.e., 97.2%, 98.6% and 90.3% of classical -, intermediate- and non-classical monocytes, respectively). In conclusion, we detected a marked IFN-α induction and observed that TLR9 agonism exerted a time-dependent effect on monocyte expression of CD169.

### TLR9 Agonist Immunomodulation in Human Hematopoietic Stem Cell-Transplanted NOG Mice

**Figure 2:**
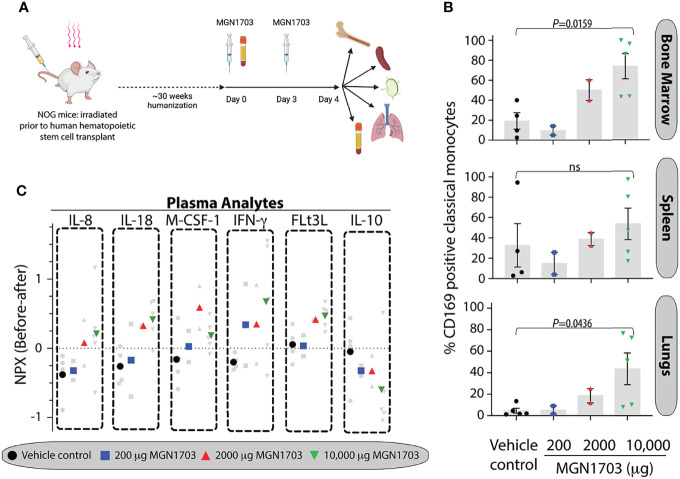
*In vivo* immunomodulatory impact on human monocytes of TLR9 agonist in humanized NOG mice. **(A)** Schematic depiction of humanized NOG mice experiment. Humanized NOG mice were dosed with MGN1703 in one of 3 different dosage groups (200, 2,000 and 10,000 μg/mouse) or with physiological saline on day 0 and day 3. Blood collection was performed at baseline and at the time of necropsy on day 4, where bone marrow, spleen, lymph nodes and lungs were collected as well. Graphic created with Biorender. **(B)** Tissue cells were isolated on day 4 from bone marrow, spleen, and lungs. These cells were evaluated for CD169 expression using flow cytometry. Percentages of CD169 positive classical monocytes are depicted in the bar graphs. Individual mice values are shown together with each bar that depicts the mean (+/- SEM). Data were analyzed by ANOVA and MGN1703 dose groups were compared with vehicle group using Dunnets multiple comparison tests. **(C)** Cytokines and chemokines in day 4 plasma were analyzed relative to baseline using O-link technology. Individual mice normalized protein expression (NPX) values are depicted as changes from baseline in light grey with the dose-group average denoted by large colored symbols.

We performed a dose-escalation study of the TLR9 agonist MGN1703 to evaluate the immunotherapy potential of this molecule in humanized NOG mice. In **Figure 2A**, an outline of the dosing schedule in mice is presented. On day 4 [after 2 doses of MGN1703 or placebo (physiological saline)], we harvested primary and secondary immune tissues from humanized NOG mice. Using flow cytometry, we determined the percentage of monocytes expressing CD169 on their surface. In the bone marrow, we observed a MGN1703 dose-dependent change in the percentage of classical monocytes exhibiting CD169 expression (19.3% CD169+ in vehicle treated compared to 73.6% in 10,000 ug MGN1703 treated mice, p=0.0159) (**Figure 2B**). Similar outcomes were observed when analyzing monocytes harvested from the spleen (32.6% CD169+ in vehicle treated to 53.4% in 10,000 ug MGN1703, not statistically significant) and lungs (4.6% CD169+ in vehicle treated to 43.5% CD169+ in 10,000 ug MGN1703, p=0.0436). Furthermore, we analyzed T cell activation in MGN1703-treated humanized NOG mice. In bone marrow, spleen, lymph nodes and lung tissues, we determined the percentage of CD4+ and CD8+ T cells expressing the early activation marker CD69. While we did not observe the same magnitude of dose-increase as seen in classical monocytes of humanized NOG mice expressing CD169, we did note that consistently elevated levels of CD4+ and CD8+ T cells expressing CD69 were found in bone marrow and spleen in mice receiving the highest does of MGN1703. This finding was not observed in lymph node and lung tissues (**Supplementary Figure 2**).

In addition to quantitating CD169 positive cells in humanized NOG mice, we also collected plasma prior to drug dosing and at time of necropsy. With these samples, we performed a comprehensive multiplex analyte investigation using O-link technology. This platform is designed to analyze 92 secreted human proteins. Of these, we censored 13 because of non-specific background detected in plasma from non-humanized NOG mice. Of the remaining 79 analytes, we found that 31 were at or below limit of detection in all mice, regardless of treatment. The 48 remaining analytes were analyzed across groups. IL-8, IL-18, M-CSF-1, IFN-γ, FLt3L, and IL-10 showed dose-dependent modulation patterns in response to MGN1703 immunotherapy (**Figure 2C**). Specifically, IL-8, IL-18, M-CSF-1 (except at the highest dose), IFN-g, and FLt3L exhibited successively higher normalized protein expression (NPX) values with escalated dosing. In contrast, IL-10 showed an inverse pattern. Overall, the humanized NOG mouse data demonstrate that, in this model, there is a MGN1703 dose effect on the percentage of CD169-expressing monocytes in both primary and secondary immune tissues. These changes were accompanied by concomitantly modulation of human chemokine and cytokine levels.

### CD169 Upregulation Following MGN1703 Dosing in Humans

**Figure 3:**
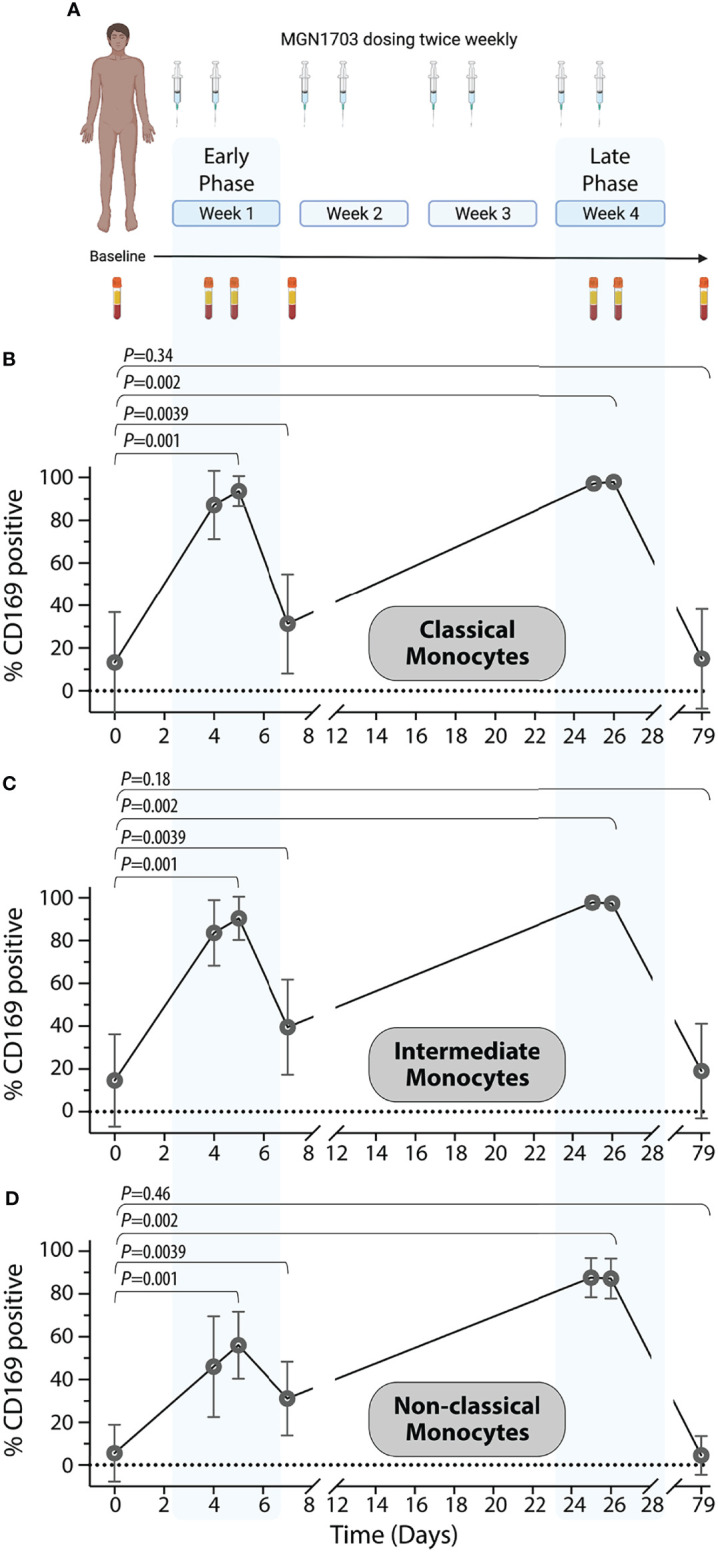
*In vivo* upregulation of CD169 on human monocytes in a clinical TLR9 agonist study. **(A)** Schematic layout of a clinical Phase I/IIa trial (clinicaltrials.gov NCT02443935) investigating the immune-enhancing effects of TLR9 agonist (MGN1703) in HIV-infected individuals. Graphic created with Biorender. **(B–D)** Percentages of classical monocyte **(B)**, intermediate monocytes **(C)**, and non-classical monocytes **(D)** expressing CD169 are shown depicted with symbol and lines for averages (+/- SEM). Blue shaded areas indicate “early” and “late” dosing windows (dose 1 + 2 in the first dosing week and dose 7 + 8 in the last dosing week). The following Wilcoxon sign-ranked test statistical comparisons were made for each monocyte population: baseline vs. day 5 (Peak 1); baseline vs. day 7 (Trough); baseline vs. day 26 (Peak 2); and baseline vs. day 79 (Follow-up).

In our previous clinical trial (NCT02443935), ART-treated HIV-infected individuals received twice weekly dosing of MGN1703 for four weeks (Vibholm et al., 2017). **Figure 3A** summarizes the study protocol relevant to this work. The figure presents an outline of the dosing as well as the PBMC sampling times used for CD169 analyses presented in **Figures 3B–D** and monocyte subset proportion data presented in **Supplementary Figure 3**. Using viably cryopreserved PBMCs, we determined the percentage of CD169 positive monocyte subsets at each time point. Overall, we identified a rapid and highly uniform increase in CD169 positive cells within all three monocyte subsets following successive MGN1703 dosing. Specifically, we observed a mean of 13.3% of classical monocytes expressed CD169 at baseline and this increased to 93.6% (p=0.001) after the 2^nd^ dose of MGN1703 (peak 1), and close to all (mean 97.9%, p=0.002) of the classical monocytes expressed CD169 after the 8^th^ dose (peak 2). These increases were mirrored in the intermediate monocyte population where the percentage of CD169+ monocytes shifted from 14.6% to 83.6% (p=0.001) and 97.3% (p=0.002) from baseline after the second (peak 1) and eighth (peak 2) MGN1703 doses, respectively. Finally, significant increases in the percentages of CD169 positive monocytes were also observed in the non-classical monocyte subset, albeit of lesser magnitude increasing from 5.6% CD169+ at baseline to 56.1% (p=0.001) after the second dose (peak 1) and 87.2% (p=0.002) after the eighth dose (peak 2). For all subsets these increases were transient and highly dynamic as samples collected on day 7 (four days after the second dose), revealed that the percentage of monocytes expressing CD169 contracted, although not back to baseline levels (p=0.039 for all subsets). The transient nature of this MGN1703-induced change in monocyte physiology was further bolstered by the fact that at the post-dosing clinical follow-up timepoint (study day 79) the percentage of monocytes expressing CD169 was indistinguishable from the baseline values. This was observed for all three monocytic subsets. Lastly, the relative distributions of the three monocytic subsets were quantified to better understand the impact of MGN1703 dosing on the peripheral blood monocyte populations (**Supplementary Figure 3**). During the course of MGN1703 dosing, limited changes in the proportions of classical monocytes were observed. In contrast, the relative levels of intermediate monocytes increase from 5.8% at baseline to 10.2% on day 5 (p=0.005) and 9.4% on day 26 (p=0.006) and after two and eight doses, respectively. Accordingly, the relative levels of the non-classical monocytes dropped from 8.3% at baseline to 6.7% (not statistically significant) by day 5 and further declined to constitute 4.0% (p=0.039) of the monocytes on day 26 within the last week of dosing. These data led us to conclude that the percentages of CD169 positive monocytes significantly increased across all monocytic subsets as a consequence of MGN1703 administration. After multiple administrations, close to all (>97%) monocytes in circulation exhibit expression of CD169. These values had returned to baseline levels within the 8 weeks from the last MGN1703 administration.

## Discussion

TLR9 immunotherapy is gaining traction for use in treating infectious diseases as well as cancers. To this point, we are currently testing MGN1703 in HIV cure trials in combination with broadly neutralizing antibodies and therapeutic vaccination (clinicaltrials.gov NCT03837756 and NCT04357821). The goal in these studies is to augment innate immune cells (e.g., improve NK cell’s ability to induce antibody dependent cell cytotoxicity) and to boost antigen processing and presentation. These outcomes are expected to lead to enhanced anti-HIV adaptive immune responses. One of the hallmarks of TLR9 agonist stimulation, and an indicator of innate immune activation, is treatment-induced IFN-α production. However, measuring IFN-α is rather challenging compared to measuring other cytokines. Thus, there is a strong need for a simple biomarker of the induced immune surveillance activity of TLR9 agonist treatments. To address this need, we assessed the impact of TLR9 agonist immunotherapy on human cells *ex vivo*, *in vivo* in humanized NOG mice, and *in vivo* in people living with HIV.

Others have shown that IFN-α induces increased CD169 expression in monocytes (Puryear et al., 2013). We first extended those findings and looked at whether TLR agonism and changes in physiological levels of IFN-α, would change the proportions of cells expressing CD169. We observed such changes in a little as 16 hours. Furthermore, these changes were consistently observed across all monocyte subsets: classical, intermediate, and non-classical. These *ex vivo* findings confirmed that TLR9 agonism using MGN1703 was sufficient to induce changes in CD169 expression on monocytes, comparable to studies using CpG-ODN 2216 to induce changes in CD169 measured in total PBMC populations (York et al., 2007). However, there are certain limitations to *ex vivo* experiments that can only be resolved by performing *in vivo* studies (e.g., organ system specific effects). Therefore, we also treated humanized NOG mice with the TLR9 agonist MGN1703 and assessed the impacts of this immunotherapy in primary and secondary immune tissues.

We chose to work in humanized NOG mice because these animals (along with other humanized mice generated *via* reconstitution with human hematopoietic stem cells) are extremely useful tools in pre-clinical analysis of drug effects on human cells *in vivo* (Denton and Garcia, 2011). They are also very powerful models for assessing drug impacts on human tumor burdens and the clearance of virus infections (Denton et al., 2016). Recent efforts include assessing the applications of different humanized mice models for the study of immunotherapy and adoptive cell therapy interventions (Yin et al., 2020). In these contexts, humanized mice have contributed with critical insights into the *in vivo* spatial and temporal effects of novel and exciting therapies (e.g., human CAR T cells targeting human tumors, including models with fully autologous cancer and CAR T cell, derived from CD34+ transplants) (Jin et al., 2019). However, the contribution of the innate immune responses in these models remains relatively unexplored, mainly because many of these models do not fully support the development of human myeloid cells (Rongvaux et al., 2014). In our recent paper, we showed that human STING activation in humanized NOG mice leads to production of human cytokines from human innate immune cells. These findings confirmed that humanized NOG mice are indeed responsive to innate immune stimulation and capable of exhibiting human innate immunity (Andersen et al., 2019). Our current work allows us to further assess the breadth of the innate responses in humanized NOG mice and specifically monitor the effect of administration of a TLR9 agonist in this model.

In humanized NOG mice, we determined that the percentage of human CD169+ classical monocytes increase in bone marrow, spleen, and lungs with increasing dose of MGN1703. These findings mirrored our *ex vivo* human data. In the plasma of the mice receiving the highest dose of MGN1703, we observed a higher induction of the chemoattractant cytokines IL-8, proinflammatory IL-18, cell maturing M-CSF-1 and FLt3L, as well as IFN-γ. Conversely, the anti-inflammatory cytokine IL-10 showed a dose-dependent reduction. These findings reflect a maturing human innate response in these animals following TLR9 agonism. To determine whether the *ex vivo* and *in vivo* humanized mouse-derived data were representative of the impacts of MGN1703 on CD169 expression in humans, we analyzed samples from our recent clinical trial using this immunotherapy in people with HIV.

In the clinical trial participants examined herein, we observed that TLR9 agonism led peripheral blood monocytes to express CD169. This effect was transient and temporally associated with immunotherapy dosing. We also noted that the levels of non-classical monocytes decreased over the course of TLR9 agonist treatment with a 50% relative reduction from baseline to after 8 doses. This observation is likely explained by the discovery that CD16+ monocytes leave circulation and differentiate to tissue dendritic cells as part of the activation and maturation process (Randolph et al., 2002; Wacleche et al., 2018). Thus, our data suggest that upregulation of CD169 in response to TLR9 agonism activates monocytes to have enhanced antigen presentation function. Such a hypothesis agrees with the beneficial effect of TLR9 agonist as vaccine adjuvants (Sogaard et al., 2010; Jensen et al., 2012). Importantly, a previous study of CD169 found a correlation with viral loads in SIV-infected macaques (Kim et al., 2015). We therefore evaluated whether the levels of viral load during latency reversal by MGN1703 impacted CD169 on the monocyte subsets in individual participants but were unable to uncover any correlations.

The proximal effector molecule of TLR9 agonist therapy is IFN-α and IFN-α causes a dose-dependent increase in the number of monocyte-derived macrophages expressing CD169 (Krieg, 2006; Saito et al., 2015; Akiyama et al., 2017). From our trial, we previously reported the plasma levels of IFN-α where we observed 12 of 15 trial participants exhibited increases in plasma IFN-α following the first week of MGN1703 dosing (Vibholm et al., 2017). The corollary to this is that plasma levels of IFN-α remained at/below the detection limit in 3 of 15 (20%) participants. Thus, plasma IFN-α levels, which are challenging to detect, is not a perfect biomarker for TLR9 agonist therapy activity. We therefore considered using the levels of CD169-expressing macrophages as a biomarker of TLR9 agonism. However, CD169 is a general activation marker for phagocytes and CD169 expression can be induced in the absence of TLR agonist immunotherapy. This is illustrated by the findings in untreated SIV and SARS-CoV-2 infections (Kim et al., 2015; Bedin et al., 2021). So, before pursuing increases in CD169 expressing macrophages as a biomarker for TLR9 agonist therapy in our clinical trial samples, we first confirmed that our aviremic clinical trial participants did not have high basal levels of CD169 expressing macrophages. Our finding that these levels were all relatively low (>10%) supporting the potential for CD169 to serve as a biomarker for TLR9 agonist activity in this patient population as hypothesized. When we looked at these levels of monocytes expressing CD169 following TLR9 agonist therapy, we found that there were marked and readily detectable increases in the percentages of monocytes expressing CD169 in 12 of 12 participants samples analyzed. Remarkably, on PBMC material obtained in the last week of dosing the shift in monocyte population expressing CD169 is almost absolute with 97% of monocytes deemed positive for CD169. Further, we observed that 100% of participants exhibited the highly elevated proportions of monocytes expressing CD169. These facts strongly support the hypothesis we tested - namely that increase in the numbers of CD169 expressing monocytes is a strong biomarker of innate immune activation by TLR9 agonism. The utility of this biomarker of innate immune activation is likely not limited to only TLR9 agonism-based immunotherapeutic strategies. To this point, two recent studies have investigated a TLR7 agonist (vesatolimod) in ART-suppressed HIV-infected individuals (Riddler et al., 2021; SenGupta et al., 2021). These reports detail increases in immune cell activation, specifically in the T cell and NK-cell compartments. They also noted an important nuance in their data, whereby there was an induction of interferon-stimulated gene transcription even in dose groups that did not exhibit detectable changes in the levels of plasma IFN-α. Based on the data presented herein, CD169 levels on monocytes could potentially illuminate the dose-relationship of TLR7 agonist bioactivity with greater resolution than IFN-α levels in such low-dose settings. Thus, it is possible that changes in CD169 expression could serve as a biomarker in future studies with TLR7 agonists also.

Because CD169 is a general activation marker for phagocytes it can be triggered in the absence of TLR agonist immunotherapy. This is illustrated by the findings in untreated SIV and SARS-CoV-2 infections (Kim et al., 2015; Bedin et al., 2021). Here, in the context of treated HIV infection, pre-TLR9 agonist therapy CD169 levels were relatively low and there was a robust and temporally-associated increase CD169 expression which strongly supports our tested hypothesis.

In HIV research, there is another perspective on CD169 that must be considered. *Ex vivo* studies as well as different mouse models have been used to examine whether CD169 can mediate trans-infection of HIV-1 (Izquierdo-Useros et al., 2012; Pino et al., 2015; Perez-Zsolt et al., 2019). These studies have highlighted that CD169 on dendritic cells can sequester viral particles, retain those viruses in endosomes, and facilitate trans-infection of activated CD4+ T cells in subsequent immunological synapses. Such observations must be considered when investigating immunotherapies, as reported here, that lead to substantial upregulation of CD169 on myeloid cells in HIV infected patients. Importantly, the effect of HIV trans-infection in humans has been investigated in naturally occurring CD169 null individuals where no HIV disease phenotypic differences were observed (Martinez-Picado et al., 2016). However, genetic observational studies such as this one need to be interpreted with caution. Furthermore, in our previous studies in HIV-infected individuals, we did not detect expanded levels of HIV-infected CD4 T cells in neither periphery nor in lymphoid tissues (Krarup et al., 2018; Vibholm et al., 2019; Vibholm et al., 2019).

When taken together, the *ex vivo* and *in vivo* data presented herein demonstrates that CD169 is a powerful and sensitive biomarker of TLR9 agonist bioactivity, easily detected after both *ex vivo* and *in vivo* induction. Given that there are ongoing and planned clinical trials using TLR agonists, and other immunotherapies that trigger IFN-α production, having the ability to look at CD169 modulation as a readily detectable biomarker will be a major asset to the clinical development of immunotherapy interventions.

## Materials and Methods

### PBMC and Cell Culture Stimulation

Human PBMCs were isolated from EDTA-stabilized venous blood from clinical trial participants or from buffy coats obtained from healthy blood bank donors for *ex vivo* stimulations. Ficoll gradient centrifugation was used to isolate PBMCs. For culture stimulations, eight million freshly isolated PBMCs were plated in each well of a 6-well plate supplemented with RPMI 1640 medium, 10% heat-inactivated FBS, 100 IU/mL Penicillin and 100 μg/mL Streptomycin. Cells were stimulated with 3 μM MGN1703 at different timepoints to obtain cells stimulated for 4, 8, 16 and 24 hours. All cells were harvested at the same time by using 10 mM EDTA (UltraPure™, Invitrogen) followed by the use of cell scrapers to ensure maximum monocyte recovery. Unstimulated cells were used as control. IFN-α was analyzed following 24 hours of stimulation. Briefly, supernatants were collected and cytokine quantification was performed using Mesoscale Discovery U-plex kits, as recommended by the manufacturer (catalogue # K151VHK) (Offersen et al., 2016).

### Humanized NOG Mice

Animal experiments were conducted under animal license 2017-15-0201-01312 approved by the Danish Animal Experiments Inspectorate. We used female 5–6 weeks old NOG (NOD.Cg-Prkdcscid Il2rgtm1Sug/JicTac; Taconic, Silkeborg, Denmark) mice. Following, irradiation with 0.75 Gy from a Cs^137^ gamma source, 14 mice received intravenous human hematopoietic stem cells from one of three human donors used. Human hematopoietic stem cells, defined by expression of hCD34, were enriched from umbilical cord blood obtained during caesarean section births of females. Umbilical cord bloods were obtained *via* anonymous donation under informed written consent from the mother. Briefly, human hematopoietic stem cells were enriched using EasySep™ Human Cord Blood CD34 Positive Selection Kit II (Stemcell, Vancouver, Canada). Human immune reconstitution in each mouse was determined using flow cytometry analysis of PBMCs as we have previously described (Andersen et al., 2019; Andersen et al., 2020; Andersen et al., 2020). 28-32 weeks post-human hematopoietic stem cell transplantation, mice with 13-44% human chimerization in peripheral blood were pairwise randomized (balancing donors and humanization percentage) into four treatment groups. Two doses of 200 μg/mouse, 2000 μg/mouse or 10,000 μg/mouse MGN1703 were delivered intraperitoneal (I.P.) three days apart, followed by necropsy on day four. Control mice were injected with physiological saline. Blood samples for plasma isolation were collected at baseline and on day 4. Mononuclear cells used for flow cytometry analyses were harvested from tissues as we previously described (Andersen et al., 2020).

### Human Clinical Trial Samples

Clinical samples for this biomarker investigation were obtained from an investigator-initiated, single-arm, open-label phase 1b/2a trial investigating twice weekly dosing with 60 mg MGN1703 for 4 weeks (Vibholm et al., 2017). Fifteen HIV-infected participants (18 years of age and older) were enrolled. Blood samples for this study were collected at baseline, and on days 4, 5, 7, 25, 26 and 42 (**Figure 3A**). The trial enrolled participants at either Aarhus University Hospital or Hvidovre Hospital, Denmark. The clinical trial was approved by the National Health Ethics Committee, Denmark (case number 1-10-72-10-15), the Danish Medicines Agency (case number 2015014125), and the Danish Data Protection Agency. Each participant provided written informed consent prior to any study procedures. Additional information on inclusion and exclusion criteria can be found at clinicaltrials.gov NCT02443935.

### Flow Cytometry

*Ex vivo* stimulated PBMCs and PBMCs from clinical trial participants were analyzed using flow cytometry to determine the percentage of monocytes expressing CD169. The PBMCs were washed with PBS and stained with LIVE/DEAD fixable NEAR IR Dead Cell Kit (Invitrogen) on ice and in the dark for 30 minutes. After two washing steps, the cell surfaces were blocked with Human TruStain FcX (Biolegend) and incubated for 10 minutes at room temperature. Subsequently, the cells were stained for 30 minutes at room temperature with a minimum of 50μL Brilliant stain buffer (BD Biosciences) and the following antibodies: CD3 (SK7) PE-Cy7 (BD Biosciences, 557851), CD14 (M5E2) BV421 (Biolegend, 301829), CD16 (3G8) PerCP-Cy5.5 (Biolegend, 302028), CD20 (2H7) BV605 (Biolegend, 302333), CD56 (NCAM16.2) BV605 (BD Biosciences, 562780) and CD169 (7–239) BB515 (BD Biosciences, 565353). The cells were washed and then analyzed on a FACSVerse™ (BD Biosciences). Data were analyzed in FlowJo v10.6.2. Paired ANOVA test with Dunnets multiple comparison was used to compared baseline levels to the different on-drug time-points.

We investigated CD69 levels on T cells and CD169 on monocytes within single cell suspensions isolated from MGN1703-treated humanized NOG mice (or control mice) organs. Cells were incubated with 5 μl Human TruStain FcX™ (Biolegend) for 10 min at room temperature, followed by 30 min incubation at room temperature with the following panel of mouse anti-human antibodies from Biolegend (BL) or BD Biosciences (BD); CD3 (BUV395, BD564001, clone SK7), CD4 (BUV496, BD564652/51, clone SK3), CD14 (BUV737, BD612763, clone M5E2), CD33 (BV605, BL366612, clone P67.6), CD197 (BV785, BL353230, clone G043H7), CD45RA (FITC, BD555488, clone HI100), CD16 (PerCP-Cy5.5,BL302028, clone 3G8), SIGLEC-1 (PE, BD565248, clone 7-239), CD45 (PE-Cy7, BL368532, clone 2D1), CD69 (APC, BL310910, clone FN50), CD8 (AlexaFlour, BL565192, clone SK1). Stained samples were washed twice with PBS containing 2% FBS, fixed with PFA (1%) for 15min at 4°C, and washed again. All samples were stored overnight at 4°C in FACS buffer, before being analyzed on a BD LSRFotessa™ flow cytometer. Data were analyzed in FlowJo v10.6.2. Gating strategies are presented in **Supplementary Figure 1**. ANOVA test followed by Dunnetts multiple comparison was used to compare vehicle-treated group to the different MGN1703 dose groups.

### O-Link Analysis

Humanized NOG mouse plasma samples were analyzed for secreted proteins using the O-link Proteomics 92-analyte Inflammation panel performed by BioXpedia (Aarhus, Denmark). Briefly, this multiplex immune-PCR assay quantifies protein levels based on oligo-labelled antibody binding. Analyte-specific antibody binding results in hybridization of complementary oligo regions, which serve as qPCR substrates giving analyte-specific Ct-values relative to the analyte concentration. The O-link NPX Manager Software converts Ct-values to NPX values on a log2 scale that are relative to a fixed correction factor and account for both intra- and inter-assay variation. Thus, NPX values are relative within each analyte. Limit of detection was determined using buffer containing no analytes. Plasma from three non-humanized NOG mice were also assessed to determine background signals in this assay. This resulted in discard of 13 analytes (NT-3, CXCL6, MMP-10, ARTN, FGF-21, MCP-4, TGF-a, SCF, LAP-TGF, OPG, STAMBP, TWEAK, DNER). The remaining analytes were analyzed by subtracting the day 0 plasma quantification from the day 4 level. This provides a change of the relative NPX-value where positive values indicate an increase during the study. Change in the MGN1703 dose groups were compared to the vehicle-treated group using a non-parametric Mann-Whitney test.

## Data Availability Statement

The original contributions presented in the study are included in the article/**Supplementary Material**. Further inquiries can be directed to the corresponding author.

## Ethics Statement

The studies involving human participants were reviewed and approved by National Health Ethics Committee, Denmark and the Danish Medicines Agency. The patients/participants provided their written informed consent to participate in this study.

## Author Contributions

Conceived and designed the study: MT, OS, SL, AA, PD. Conducted experiments: SL, MP, IM, AA. Analyzed data and contributed samples: MT, OS, SL, AA, MP, LV, LØ, AM, PD. Obtained funding: MT, OS, PD. Wrote the manuscript: MT, PD. Edited and approved the manuscript: SL, MP, IJ, AM, OS, AA, PD, MT. All authors contributed to the article and approved the submitted version.

## Funding

This study was supported by a grant from the Independent Research Fund Denmark to M. Tolstrup (Grant number #9039-00039B) and by a grant from the NovoNordisk Foundation to M. Tolstrup (Grant number #NNF17OC0028462). SSFL was supported by a PhD scholarship from Aarhus University.

## Conflict of Interest

The authors declare that the research was conducted in the absence of any commercial or financial relationships that could be construed as a potential conflict of interest.

## Publisher’s Note

All claims expressed in this article are solely those of the authors and do not necessarily represent those of their affiliated organizations, or those of the publisher, the editors and the reviewers. Any product that may be evaluated in this article, or claim that may be made by its manufacturer, is not guaranteed or endorsed by the publisher.
