# Supplementary material for: CD169 (Siglec-1) as a Robust Human Cell Biomarker of Toll-Like Receptor 9 Agonist Immunotherapy
Source: Front Cell Infect Microbiol. 2022 Jul 5;12:919097. doi: 10.3389/fcimb.2022.919097 (PMC9294151; doi:10.3389/fcimb.2022.919097)
Supplement: Supplementary file 1 [file DataSheet_1.pdf]

Supplemental Figure 1

Gating Strategy for Humanized Mouse Analyses

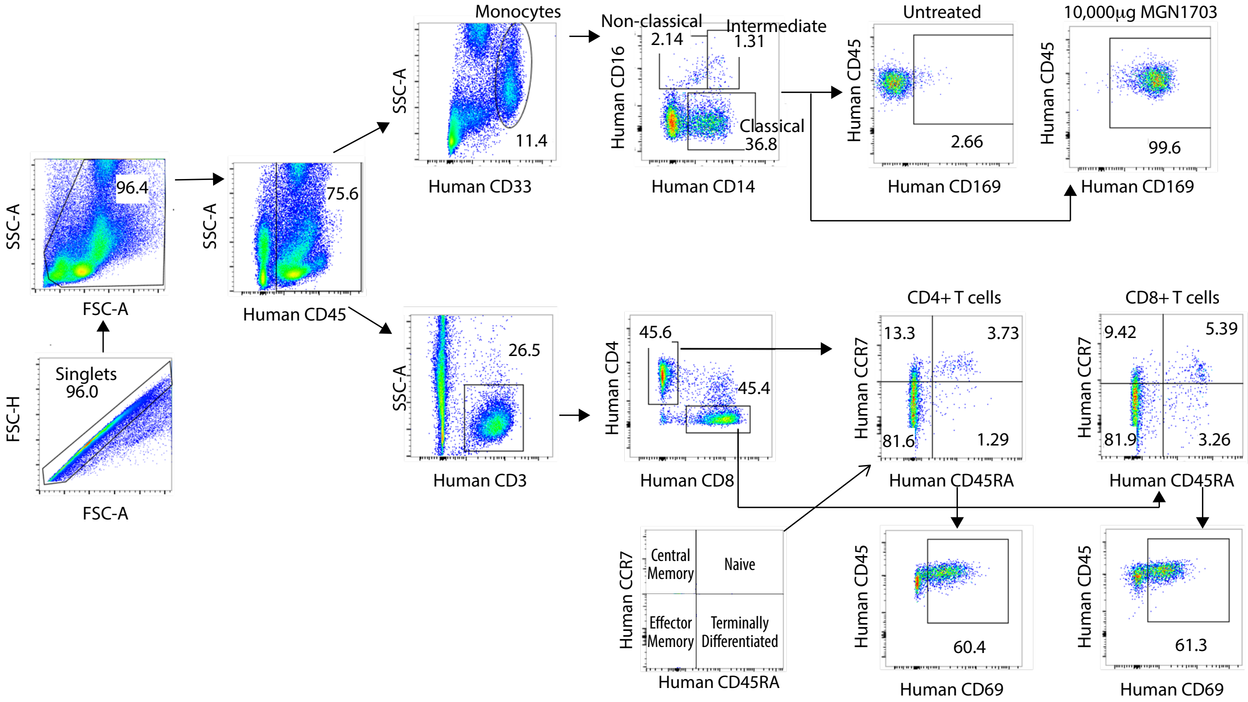

Supplemental Figure 1: Gating strategy for humanized mouse analyses. This graphic depicts the gating strategy followed for analyses of human monocyte subsets as well as human T cell subsets. Bone marrow is the tissue shown. The depiction starts with singlet discrimination. Once human cells were gated, the strategy bifurcates. Across the top, monocyte subsets are followed through assessment of CD169 expression. Classical monocytes are depicted in the CD169 plots. Across the bottom, T cell subsets are followed through assessment of CD69 expression. Effector memory T cells are depicted in the CD69 plots.

Supplemental Figure 2

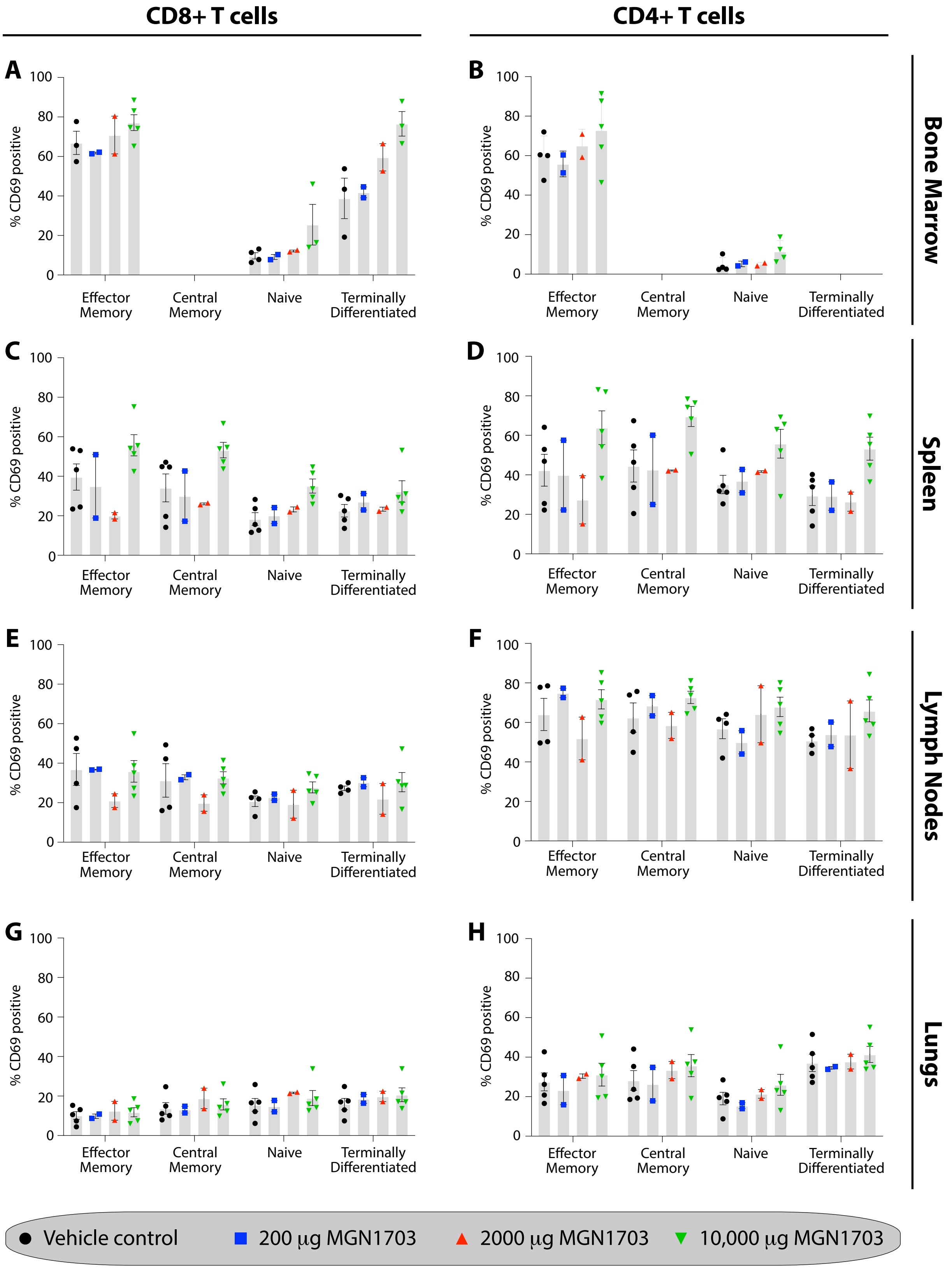

Supplemental Figure 2: *In vivo* immunomodulatory impact on human T cells of TLR9 agonist in humanized NOG mice. A-H) Tissue cells were isolated on day 4 from bone marrow (A-B), spleen (C-D), lymph nodes (E-F), and lungs (G-H). These cells were evaluated for CD69 expression using flow cytometry. Percentages of CD69 positive CD8+ T cell subsets (left column) and CD4+ T cell subsets (right column) are depicted in the bar graphs. Individual mice values are shown together with each bar that depicts the mean (+/- SEM). The legend indicates the symbol associated with each treatment category.

Supplemental Figure 3

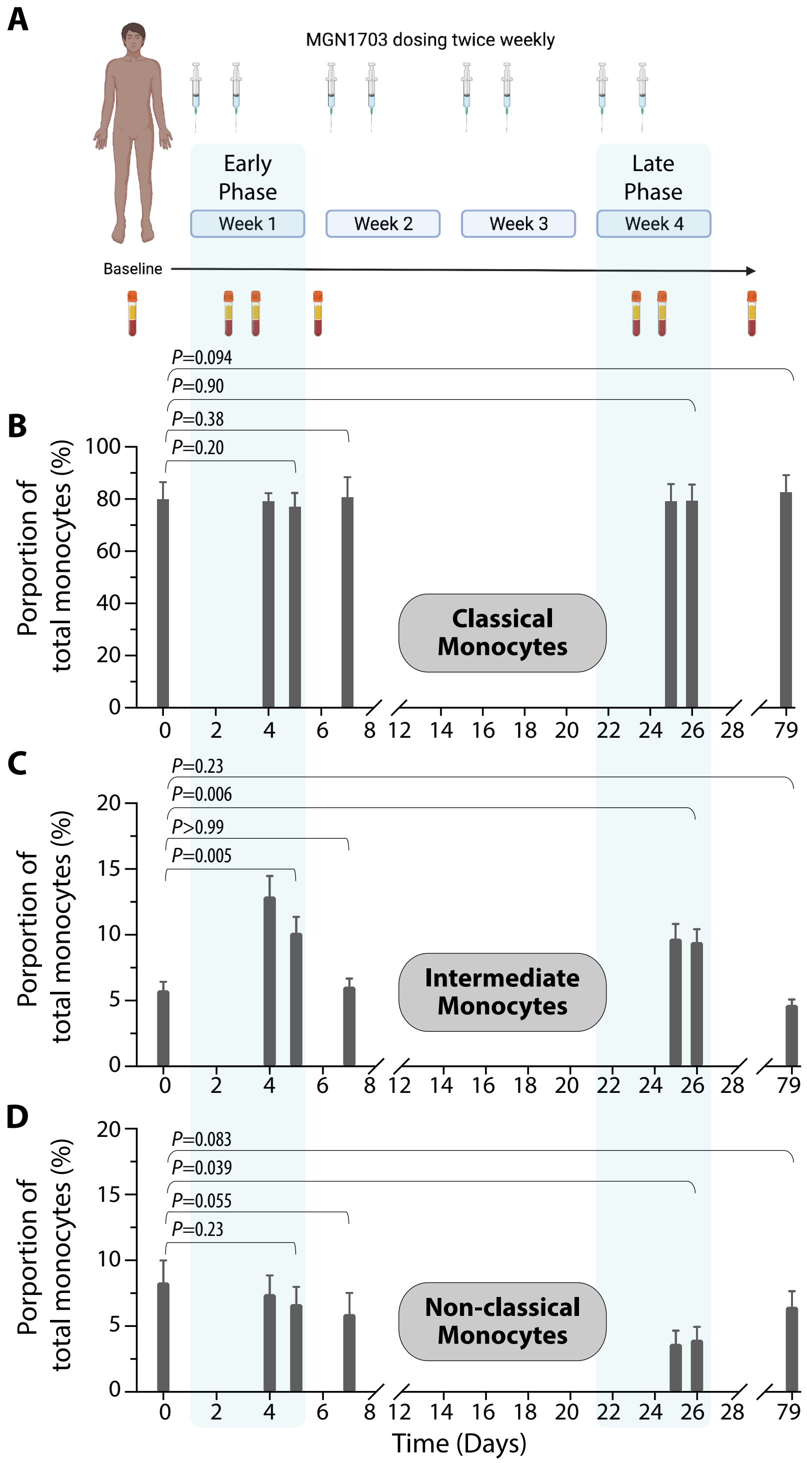

Supplemental Figure 3: *In vivo* proportions of human monocyte subsets in a clinical TLR9 agonist study. A) Schematic layout of a clinical Phase I/IIa trial (clinicaltrials.gov NCT02443935) investigating the immune-enhancing effects of TLR9 agonist (MGN1703) in HIV-infected individuals. Graphic created with Biorender. B-D) Proportion of total monocytes that is classical monocyte (B), intermediate monocytes (C), or non-classical monocytes (D) is depicted in the bar-graphs with mean (+/- SEM) based on 13-15 individuals. Blue shaded areas indicate "early" and "late" dosing windows (dose 1+2 in the first dosing week and dose 7+8 in the last dosing week). The following Wilcoxon sign-ranked test statistical comparisons were made for each monocyte population: baseline vs. day 5 (Peak 1 from Fig. 3); baseline vs. day 7 (Trough from Fig. 3); baseline vs. day 26 (Peak 2 from Fig. 3); and baseline vs. day 79 (Follow-up).
